# Design and development of an efficient RLNet prediction model for deepfake video detection

**DOI:** 10.3389/fdata.2025.1569147

**Published:** 2025-07-09

**Authors:** Varad Bhandarkawthekar, T. M. Navamani, Rishabh Sharma, K. Shyamala

**Affiliations:** School of Computer Science and Engineering (SCOPE), Vellore Institute of Technology (VIT), Vellore, Tamil Nadu, India

**Keywords:** ResNet, Long Short Term Memory Networks (LSTM), deep learning, deepfake detection, explainable artificial intelligence

## Abstract

**Introduction:**

The widespread emergence of deepfake videos presents substantial challenges to the security and authenticity of digital content, necessitating robust detection methods. Deepfake detection remains challenging due to the increasing sophistication of forgery techniques. While existing methods often focus on spatial features, they may overlook crucial temporal information distinguishing real from fake content and need to investigate several other Convolutional Neural Network architectures on video-based deep fake datasets.

**Methods:**

This study introduces an RLNet deep learning framework that utilizes ResNet and Long Short Term Memory (LSTM) networks for high-precision deepfake video detection. The key objective is exploiting spatial and temporal features to discern manipulated content accurately. The proposed approach starts with preprocessing a diverse dataset with authentic and deepfake videos. The ResNet component captures intricate spatial anomalies at the frame level, identifying subtle manipulations. Concurrently, the LSTM network analyzes temporal inconsistencies across video sequences, detecting dynamic irregularities that signify deepfake content.

**Results and discussion:**

Experimental results demonstrate the effectiveness of the combined ResNet and LSTM approach, showing an accuracy of 95.2% and superior detection capabilities compared to existing methods like EfficientNet and Recurrent Neural Networks (RNN). The framework's ability to handle various deepfake techniques and compression levels highlights its versatility and robustness. This research significantly contributes to digital media forensics by providing an advanced tool for detecting deepfake videos, enhancing digital content's security and integrity. The efficacy and resilience of the proposed system are evidenced by deepfake detection, while our visualization-based interpretability provides insights into our model.

## 1 Introduction

The proliferation of deepfake technology has introduced significant challenges to the integrity of digital content shared online. Deepfake videos, capable of seamlessly manipulating facial expressions, gestures, and voices, blur the line between reality and fiction, posing a formidable threat to the credibility of online platforms. This study introduces a new deepfake detection methodology combining temporal and spatial feature analysis to improve accuracy and resilience against sophisticated manipulation methods. The proposed method addresses key shortcomings in current detection techniques, which use Long Short-Term Memory (LSTM) networks for temporal modeling and Convolutional Neural Networks (CNNs) for spatial feature extraction. Attention-based fusion procedures are included to improve feature representation further, and explainability strategies increase model transparency, which promotes confidence in automated detection systems. This study advances the field by creating a more robust and interpretable deepfake detection model that can adjust to changing synthetic media creation methods.

This ambiguity compromises the trustworthiness of multimedia content and presents significant risks, such as the spread of misinformation, manipulation of public discourse, and even influencing elections. The ease with which individuals can generate these deceptive videos with minimal technical expertise further exacerbates the issue, making it imperative to develop robust detection methods to mitigate their impact (Zhao et al., 2023).

Current research in deepfake detection primarily focuses on analyzing spatial features, such as pixel-level inconsistencies and texture anomalies. However, these methods often fail to capture the temporal dynamics crucial for identifying deepfakes. For instance, unnatural transitions or face movements over time can reveal manipulations that spatial analysis alone might miss (Bansal et al., 2023). This gap highlights the need for approaches integrating spatial and temporal features to enhance detection accuracy and robustness across various deepfake creation techniques and compression levels (Thai et al., 2024). Furthermore, existing solutions often lack transparency, which limits users' trust in automated detection tools, particularly when understanding how these models make decisions (Elpeltagy et al., 2023).

Vashishtha et al. (2024) devised an ensemble deep learning system to distinguish between authentic and counterfeit photos. They provide a unique strategy utilizing the suggested optical flow technique, which extracts the apparent motion of image pixels, yielding more accurate findings than existing state-of-the-art methods. However, there is a lack of transparency in deepfake detection. Suratkar and Kazi (2023) introduced an innovative methodology to detect counterfeit videos. It employs transfer learning in autoencoders and a hybrid architecture of Convolutional Neural Networks (CNN) and Recurrent Neural Networks (RNN). The authors conducted the investigation using three datasets and plan to investigate additional deep learning models with minimized parameters for video-based Deepfake detection.

Soudy et al. (2024) presented a Deep Learning (DL) methodology for detecting deepfakes. The system consists of three components: Preprocessing, Detection, and Prediction. Preprocessing encompasses frame extraction, facial detection, alignment, and feature trimming. Convolutional Neural Networks (CNNs) identify ocular and nasal features. The requirement requires substantial computational resources for training and inference. Furthermore, the method may be ineffective in identifying deepfakes that alter facial regions outside the eyes, nose, and overall face. Future studies may concentrate on inventing methodologies that necessitate reduced data while preserving elevated accuracy levels.

The growing sophistication of deepfake technology and its potential for malicious misuse, such as spreading disinformation and undermining public trust, motivates the development of more advanced detection systems. The limitations of existing spatial-focused models and the increasing quality and prevalence of deepfakes demand solutions that accurately capture videos' spatial and temporal characteristics. Moreover, there is a need for detection systems that can explain their decisions, helping users understand why certain content is flagged as manipulated, thereby enhancing trust in the technology. This study addresses the identified gaps by proposing a novel deepfake detection framework that integrates ResNet for spatial analysis and Long Short-Term Memory (LSTM) networks for temporal analysis. Although other deepfake detection methods have been investigated in existing research works, there is still a significant research gap in creating a model that successfully combines temporal and spatial information while maintaining interpretability. Existing methods either concentrate on temporal patterns, which may not be sufficient to detect subtle manipulations in high-quality deepfakes, or spatial inconsistencies, which are vulnerable to adversarial assaults. By putting forward a unique deep learning framework that combines recurrent architectures, including Long Short-Term Memory (LSTM) networks for temporal analysis and Convolutional Neural Networks (CNNs) for spatial feature extraction, this research seeks to overcome these constraints. In contrast to traditional strategies, our method uses attention-based feature fusion to improve the ability to distinguish between actual and synthetic information while maintaining resilience against different compression errors and deepfake production techniques. Additionally, the proposed approach uses explainability methods to increase openness and user confidence, making model choices easier to comprehend. This work advances dependable and trustworthy deepfake detection methods by bridging the gap between interpretability and accuracy.

The main contributions of this study are as follows.

- We present a RLNet (Resnet and LSTM) deepfake detection method incorporating ResNet for spatial analysis and Long Short Term Memory networks for temporal abnormalities, significantly enhancing detection accuracy.
- This study tackles the issues presented by various deepfake generation methods and video compression artifacts, guaranteeing the model's resilience and adaptability in practical applications.
- A comparison of existing CNN architectures and pre-trained models to identify the most effective approach for video-based deepfake detection.
- Comprehensive experimentation confirms the system's efficacy. It exhibits enhanced detection accuracy relative to current approaches and highlights its practical application.
- A focus on explainability through visualization-based interpretability, providing transparency into the model's decision-making process.

The remaining sections of this study are organized as follows: Section II comprehensively reviews the existing literature on deep fake detection using deep learning techniques. Section III describes the dataset and presents the proposed methodology of the study. Section IV presents the feature extraction, model training, and model evaluation results for the proposed model. Section V summarizes the study's main findings and provides concluding remarks regarding the proposed RLNet model for deepfake video detection.

## 2 Related works

Previous research in deepfake video detection has explored various approaches, including CNN-based feature extraction, RNN-based temporal analysis, and GAN-based adversarial training. Some studies have incorporated attention mechanisms, Graph Neural Networks, and self-supervised learning to enhance detection robustness and generalization. This study aims to build upon existing work, leveraging advancements in deep learning to develop a more accurate and robust deepfake detection system using DL techniques like LSTM and ResNet. Recent research has progressed beyond frame-level analysis by integrating temporal information. Recurrent Neural Networks (RNNs) have been investigated for this objective, yielding differing levels of success. Elhassan et al. (2022) proposed a method that examines oral motions to identify discrepancies; however, their technique is confined to particular face areas and interpretability. Elpeltagy et al. (2023) enhanced the deepfake concept by integrating audio-visual data, augmenting detecting capabilities. Nonetheless, research predominantly concentrated on domain-specific anomalies, neglecting the exploration of integrating CNNs and RNNs for a more universally applicable approach.

Khan and Dai (2021) proposed a video transformer model, demonstrating the potential of transformers for video-based deepfake detection. This technique provides incremental learning but lacks a customized architecture for deepfake detection, leading to performance constraints relative to conventional CNN-based systems. Heo et al. (2023) further enhanced this by including vision transformers with local and global feature extraction; however, they did not investigate the possible union between CNNs and temporal models such as LSTM. A significant shortcoming in current deepfake detection techniques is the absence of comparative analysis of several pre-trained models and CNN architectures, including EfficientNet, Xception, and ResNet. Furthermore, several of these studies fail to consider the interpretability of their models. In deepfake detection, ensuring openness using eXplainable AI (XAI) methodologies is crucial for fostering confidence in automated systems. Gao et al. (2024) contributed to the advancement of deepfake detection by enhancing the performance of detection methods for deepfakes created with high compression. Their research addressed the practical challenge of acquiring high-quality, uncompressed data and the associated computational costs of supervised learning based on such data. By developing techniques to mitigate these challenges, they aimed to improve the accuracy and efficiency of deepfake detection in real-world scenarios. Rafique et al. (2023) proposed an automated method for classifying deepfake images, leveraging Deep Learning and Machine Learning techniques. Their approach aimed to enhance the automated detection and classification of deepfake photos, contributing to the ongoing efforts to combat the proliferation of manipulated visual content online.

Bray et al. (2023) researched and evaluated the human ability to identify deepfake images of human faces among non-deepfake images. Their study aimed to assess the effectiveness of interventions to improve human detection accuracy, providing valuable insights into the human factors involved in detecting manipulated visual content. Malik et al. (2023) contributed significantly to deepfake detection by proposing a novel method that leverages datasets such as the Deep Fake Detection Challenge (DFDC) and Face Forensic datasets. Their research underscores the importance of utilizing diverse and comprehensive datasets to effectively train detection models, enhancing their ability to discern between authentic and manipulated videos accurately. Lin et al. (2023) introduced a CNN-based deepfake detection method incorporating multi-scale convolution and vision transformer techniques. By integrating these advanced methodologies, their approach demonstrates the potential for improving the accuracy and efficiency of deep fake detection systems, further highlighting the advancements made possible through deep learning techniques.

Kumar et al. (2024) introduced the DFN (Deep Fake Network) model architecture, representing a holistic approach to deepfake video detection. By integrating various components such as mobNet blocks, separable convolution layers, and XGBoost classifier, their model offers a comprehensive solution for identifying manipulated videos, showcasing the versatility and effectiveness of deep learning-based approaches in combating deepfake proliferation. Collectively, these studies underscore the pivotal role of deep learning in the ongoing efforts to detect and mitigate the spread of deepfake videos. By leveraging advanced techniques and innovative methodologies, researchers continue to push the boundaries of deepfake detection, highlighting the significance of ongoing research and development in this critical area. The investigation of deepfake video detection uncovers a swiftly changing environment marked by various novel methods utilizing sophisticated deep learning technologies. These approaches include many tactics, such as feature extraction using convolutional neural networks and temporal analysis using recurrent neural networks, emphasizing the need to identify spatial and temporal abnormalities in altered information. However, issues, including reliance on dataset quality, poor generalization among modification approaches, and false positives, still exist. Reducing detection errors and improving model resilience should be the main goals of future developments. Combining hybrid facial landmarks and innovative heart rate features, Farooq et al. (2025) presented a unified system that improves deepfake detection and achieves strong performance using a lightweight XGBoost classifier. It maintains competitive accuracy while providing better interpretability than deep learning-based methods. Nevertheless, there are still issues with deepfake creation methods and generalization across various datasets. Adeosun et al. (2025) showed that deepfake detection algorithms are susceptible to adversarial assaults, as shown by the notable declines in accuracy under FGSM perturbations. Techniques for preprocessing and adversarial training strengthen the model's resistance to assaults. The trade-off between adversarial resilience and clean data correctness remains a significant obstacle to practical implementation. Qadir et al. (2024) introduced ResNet-Swish-BiLSTM, a hybrid deep learning model for deepfake detection that shows promise in forensic applications with an accuracy of 96.23%. Unlike conventional approaches, it focuses on identifying artifacts across various deepfake modification techniques. Future advancements in temporal pattern analysis and reasoning are necessary since the model has difficulty identifying temporal irregularities over time. Cunha et al. (2024) used a hybrid EfficientNet-GRU network and PSO-based hyperparameter selection to improve deepfake detection, beating traditional search approaches across several datasets. Combining transfer learning and reinforcement learning-based PSO increases classification accuracy and model optimization. However, issues remain in avoiding early convergence in PSO and guaranteeing resilience against emerging deepfake-generating methods.

Incorporating many modalities and attention processes improves the resilience and precision of detection systems. Confronting practical obstacles, such as elevated compression rates and the necessity for varied training datasets, is essential for guaranteeing successful performance in real-world applications. The continual progress in deepfake detection highlights the necessity for constant study and innovation to counteract the spread of altered media and maintain the integrity of digital information. To resolve the concerns, we provide a deep learning framework that employs ResNet Convolutional Neural Networks (CNNs) and Long Short-Term Memory (LSTM) networks for accurate deepfake video detection.

## 3 Materials and methods

**Figure 1:**
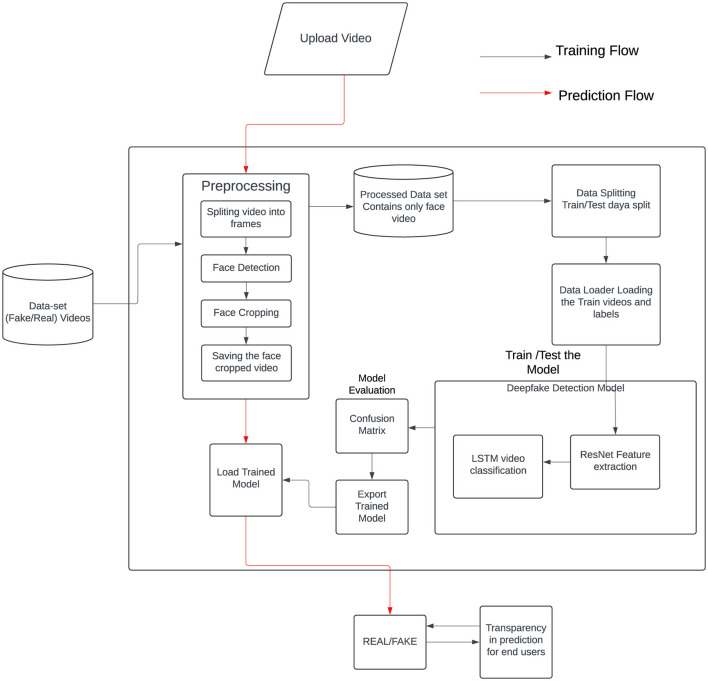
Overview of the proposed system.

The proposed system for RLNet (ResNet and LSTM) deepfake detection operates through two primary flows: training and prediction. Figure 1 shows the architectural diagram of the proposed system. In the training flow, users upload videos that will be used to train the deepfake detection model. These videos undergo preprocessing, where they are split into individual frames. In some cases, face detection and cropping are performed on these frames to focus on the regions of interest. The resulting frames are then divided into training and testing datasets to facilitate model evaluation. The training data is subsequently loaded into a deep learning model that combines Long Short-Term Memory (LSTM) networks and Residual Networks (ResNet).

Figure 1 shows the integral components of a deep learning pipeline setup, primarily validating the dataset for model training. LSTM networks are adept at handling sequential data, making them suitable for video analysis, while ResNet is known for its robust feature extraction capabilities through residual learning. Combining these architectures allows the model to classify video frames as real or fake. In summary, the system efficiently trains a deep learning model for deepfake detection by preprocessing videos, dividing data, training the model, and evaluating its performance. The trained model is then utilized in a prediction workflow to assess the authenticity of new videos, ensuring robust and accurate deepfake detection.

### 3.1 Dataset description

The deepfake detection dataset is designed to aid in developing and evaluating deep learning models for identifying deepfake videos, which are artificially manipulated to alter the face or voice of a person using AI techniques. The dataset comprises genuine and manipulated videos from a public platform named Kaggle. It is organized into training and testing directories, with subdirectories for real and fake videos. Each video is labeled with a unique identifier and stored in a standardized format (e.g., MP4). Accompanying the videos is a metadata file containing information such as video ID, label (real or fake), source, duration, resolution, frame rate, and creation date. Some videos include frame-level annotations and bounding boxes indicating faces to aid region-based analysis. The dataset is intended for training and testing deep learning models, with recommended preprocessing steps, including face detection, normalization, and data augmentation.

### 3.2 Preprocessing and model training

Users initiate the deepfake detection process by uploading videos to the system. This step serves as the entry point for analysis, allowing the system to receive multimedia content for scrutiny. Uploaded videos may vary in length and content, encompassing a diverse range of visual data for assessment. Upon upload, the video undergoes preprocessing to prepare for analysis. This involves splitting the video into individual frames, a crucial step in extracting relevant information for subsequent analysis. Additionally, preprocessing may include face detection and cropping to isolate facial regions commonly targeted in deepfake manipulations. By segmenting the video and focusing on critical areas, preprocessing lays the groundwork for more accurate detection. Preprocessed video frames are partitioned into two sets: training and testing data. The training data instructs the model to distinguish between authentic and manipulated frames, providing the foundation for learning. Meanwhile, the testing data remains segregated to evaluate the model's performance independently, ensuring an unbiased assessment of its effectiveness.

#### 3.2.1 Customized ResNet

ResNet plays a crucial role in deepfake detection by extracting spatial information from individual video frames. Deepfake videos often contain subtle distortions or inconsistencies, such as irregularities in lighting, texture, or facial movements, which may go unnoticed by human observers. ResNet addresses this challenge by identifying these minor anomalies through its multi-layered neural network. The architecture's residual connections also help overcome the vanishing gradient problem, enabling deeper networks to learn more complex features without performance loss. ResNet generates a feature map for each video frame, highlighting potential spatial manipulations or abnormalities (He et al., 2019). ResNet introduces residual learning, which can be mathematically represented as:


(1) $$\begin{array}{l} y=F\left(x,\left\{W_{i}\right\}\right)+x \end{array}$$

where: *x* is the input to the residual block. *F*(*x*, {*W*_*i*_}) is the residual mapping, defined by convolutions, activations, and batch normalization. {*W*_*i*_} are the weights of the convolutional filters. *y* is the residual block output, combining the original input *x* and the result of the residual mapping.

In deepfake detection, ResNet processes each video frame and outputs a feature map for each frame *i* with dimensions:


(2) $$\begin{array}{l} f_{i}\in {\mathbb{R}}^{C\times H\times W} \end{array}$$

where: *C* is the number of feature channels. *H* and *W* are the spatial dimensions.

#### 3.2.2 LSTM video classification

The LSTM generates a classification prediction for the entire video sequence based on its identified temporal patterns. The key innovation in this approach lies in integrating both spatial and temporal feature extraction. ResNet pinpoints frame-level anomalies, while LSTM detects irregularities in the sequence of frames, significantly improving the accuracy of deepfake detection (Yu et al., 2021). This hybrid framework is designed to generalize across different deepfake generation techniques, making it applicable in real-world settings where deepfakes are created using various methods and tools. The combination of ResNet and LSTM demonstrates superior detection performance compared to models that rely solely on CNNs or RNNs, positioning it as a leading solution for deepfake detection. By leveraging both spatial and temporal data, this system offers a comprehensive approach to identifying subtle manipulations that other techniques might miss, enhancing the security and integrity of digital media. The LSTM unit operates using the following equations (Remesh et al., 2022) at each timestep *t*:


(3) $$\begin{array}{ll} f_{t} & =\sigma \left(W_{f}\left[h_{t-1},x_{t}\right]+b_{f}\right) \end{array}$$

(4) $$\begin{array}{ll} i_{t} & =\sigma \left(W_{i}\left[h_{t-1},x_{t}\right]+b_{i}\right) \end{array}$$

(5) $$\begin{array}{ll} C_{t}^{\prime } & =\tanh\left(W_{C}\left[h_{t-1},x_{t}\right]+b_{C}\right) \end{array}$$

(6) $$\begin{array}{ll} C_{t} & =f_{t}C_{t-1}+i_{t}C_{t}^{\prime } \end{array}$$

(7) $$\begin{array}{ll} o_{t} & =\sigma \left(W_{o}\left[h_{t-1},x_{t}\right]+b_{o}\right) \end{array}$$

(8) $$\begin{array}{ll} h_{t} & =o_{t}\tanh\left(C_{t}\right) \end{array}$$

Where:

- *x*_*t*_ is the input (feature map from ResNet for frame *t*).
- *h*_*t*−1_ is the hidden state from the previous timestep.
- *C*_*t*_ is the cell state.
- *W*_*f*_, *W*_*i*_, *W*_*C*_, *W*_*o*_ are weight matrices, and *b*_*f*_, *b*_*i*_, *b*_*C*_, *b*_*o*_ are biases.

LSTM, a Recurrent Neural Network (RNN), is well-suited for handling time-series data like videos, as it captures temporal interdependencies between video frames. While ResNet focuses on identifying spatial abnormalities at the individual frame level, LSTM detects inconsistencies across multiple frames. Manipulated videos often exhibit unusual temporal dynamics, such as erratic eye blinks, mismatched lip movements, or unnatural evolution of facial expressions over time. By processing the sequence of feature maps produced by ResNet, the LSTM learns the typical temporal progression of these frames and flags any deviations from this normal behavior as potential deepfake content.

### 3.3 Training and loss function

This study used the cross-entropy loss function to assess the efficacy of the deepfake detection model. This function quantifies the divergence between the expected probability and the actual labels. A reduced cross-entropy loss signifies an improved model fit, indicating greater accuracy in the model's predictions regarding classifying a video as a deepfake. In deepfake detection, the true labels are binary (1 for deepfake, 0 for legitimate), whereas the predicted probabilities indicate the model's confidence in its classification. The cross-entropy loss accurately measures the model's capacity to distinguish between authentic and altered material, serving as a significant indicator for evaluating its performance.

For binary classification, the cross-entropy loss (MEGANATHAN and KRISHNAN, 2023) is defined as:


(9) $$\begin{array}{l} L=-\frac{1}{N}\sum \left[y_{i}\log\left(p_{i}\right)+\left(1-y_{i}\right)\log\left(1-p_{i}\right)\right] \end{array}$$

Where:

- *y*_*i*_ is the actual label (1 for deepfake, 0 for authentic).
- *p*_*i*_ is the predicted probability.
- *N* is the total number of samples in the batch.

### 3.4 Model evaluation

During model evaluation, the trained deepfake detection model undergoes rigorous scrutiny to assess its performance and reliability. The cornerstone of this evaluation process is the confusion matrix. Tabular representation compares the model's predictions against the ground truth labels (i.e., whether each frame is classified as natural or fake) for the testing data set. The confusion matrix provides detailed insights into the model's performance, enabling the calculation of various performance metrics such as accuracy, precision, recall, and F1-score. Accuracy measures the overall correctness of the model's predictions, while precision quantifies the proportion of accurate optimistic predictions among all positive predictions made by the model. Conversely, Recall measures the proportion of correct optimistic predictions among all actual positive instances in the dataset. Finally, the F1-score provides a balanced measure of the model's precision and recall, offering a single metric to gauge its effectiveness in detecting deepfakes (Hariprasad et al., 2023). Upon satisfactory evaluation, the trained deepfake detection model is exported for future deployment in the prediction flow. This process ensures that the model and its learned parameters and configurations can be efficiently utilized for real-time predictions on new videos. Exporting the trained model makes it readily available for deployment in various environments and applications, streamlining the integration process into existing systems or platforms. Additionally, exporting the model allows for scalability and reusability, enabling seamless deployment across different devices or platforms to meet the diverse needs of users. Overall, the exportation of the trained model marks the culmination of the training process, transforming it into a practical tool for detecting deepfakes in real-world scenarios. The following performance metrics are used:


(10) $$\begin{array}{ll} \text{Accuracy} & =\frac{TP+TN}{\text{Total Samples}} \end{array}$$

(11) $$\begin{array}{ll} \text{Precision} & =\frac{TP}{TP+FP} \end{array}$$

(12) $$\begin{array}{ll} \text{Recall} & =\frac{TP}{TP+FN} \end{array}$$

(13) $$\begin{array}{ll} \text{F1 Score} & =2\frac{\text{Precision}\;\times \;\text{Recall}}{\text{Precision}\;+\;\text{Recall}} \end{array}$$

A confusion matrix is a table commonly used to evaluate the efficacy of a classification model by contrasting the actual and predicted classes for a given set of test data. It comprises four metrics: True Positives (TP), False Positives (FP), True Negatives (TN), and False Negatives (FN). The proposed approach is evaluated using accuracy, precision, recall, and the F1-score. The metrics utilized to assess our models are derived from Equations 10–13. The subsequent Algorithm 1 delineates the procedure:

**Algorithm 1:**
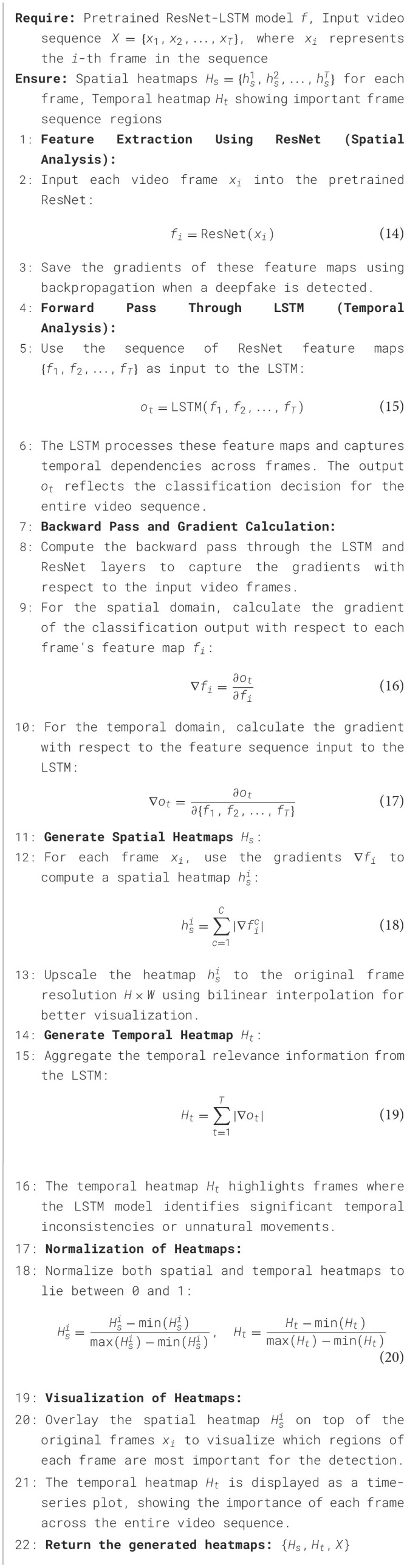
Visualization-based interpretability for ResNet-LSTM deepfake detection.

### 3.5 Testing and prediction

The system leverages the pre-trained deepfake detection model, previously trained on a dataset containing genuine and manipulated videos. This pre-trained model is equipped with the knowledge and patterns learned during training, enabling it to make predictions on new, unseen videos. The prediction begins by loading the pre-trained model into the system, ensuring it is ready to analyze incoming video data. A new video is uploaded for analysis, and the loaded model systematically analyzes the video frames, scrutinizing them for patterns indicative of deepfake manipulation. Based on the learned patterns, the model predicts whether each frame is real or fake. These individual frame predictions are aggregated to determine the overall authenticity of the video. Through this prediction flow, the system provides users with valuable insights into the authenticity of multimedia content, empowering them to make informed decisions and take appropriate actions based on the detected presence of deepfakes.

### 3.6 Model interpretability

Model interpretation is crucial for comprehending the decision-making processes of deep models. In Deepfake video detection, the synthetic frames exhibit high realism, rendering them indistinguishable from the human eye. Therefore, it is essential to examine the interpretability to comprehend how our model formulates judgments. Using the deconstructed self-attention mechanism, we can analyze our model in temporal and spatial dimensions by displaying the discriminative and salient regions (Selvaraju et al., 2017). The preprocessed image is fed to the proposed model, and a grad cam is used for explainability. Grad-CAM (Gradient-weighted Class Activation Mapping) is a method employed to show the regions of an image most significant for a neural network's classification decision. It emphasizes the areas that most significantly influence the network's decision-making process.

## 4 Results and discussion

The deepfake detection system involves developing software modules designed to address specific stages within the training and prediction flows. The Video Upload Module facilitates the seamless upload of videos, while the Preprocessing Module extracts individual frames and converts continuous video streams into discrete images for analysis. The Data Splitting Module divides processed frames into training and testing datasets, while the Data Loading Module loads training data into the deep learning model. The Model Training phase leverages LSTM and ResNet architectures, allowing the model to learn complex patterns and dependencies. The Model Evaluation Module assesses the model's performance using metrics from the confusion matrix. The Model Export Module saves the validated model for future use. The Model Loading Module prepares the pre-trained model for analysis, while the New Video Upload Module facilitates uploading new videos for analysis. The Frame Processing Module splits new videos into frames, and the Prediction Module analyzes each frame to predict its authenticity. The system's modular architecture ensures scalability, adaptability, and ethical considerations, making it an essential tool for countering deepfake proliferation and digital information integrity.

In deepfake detection, combining the strengths of Convolutional Neural Networks (CNNs) like ResNet with Recurrent Neural Networks (RNNs) such as LSTM offers a robust solution. This approach leverages ResNet for extracting detailed spatial features from individual frames and LSTM for capturing temporal dependencies across video sequences. Utilizing the dataset, which comprises both natural and manipulated videos, we evaluated the effectiveness of this hybrid model. During the experimental setup, frames were extracted from videos, resized, and normalized. ResNet50, a 50-layer deep residual network pre-trained on ImageNet, was fine-tuned to extract features from these frames. The LSTM network then processed these feature sequences to identify temporal inconsistencies typical of deepfake videos. The hybrid ResNet50 and LSTM models demonstrated high performance, as shown in Table 1. This indicates that ResNet effectively captures spatial artifacts, while LSTM successfully identifies temporal anomalies, making the combined model highly effective at distinguishing between real and fake videos. The results highlight the model's ability to generalize to unseen data, suggesting its robustness against various deepfake manipulations. This success is attributed to the complementary strengths of ResNet and LSTM, which comprehensively analyze videos' visual and temporal aspects.

**Table 1:** Performance metrics comparison of different models.

**Model**	**Accuracy**	**Precision**	**Recall**	**F1-Score**
Proposed ResNet and LSTM	95.2%	93.8%	96.0%	94.9%
EfficientNet and LSTM	92.3%	91.7%	93.0%	92.7%
ResNet and RNN	91.4%	91.2%	92.5%	92.0%

**Figure 2:**
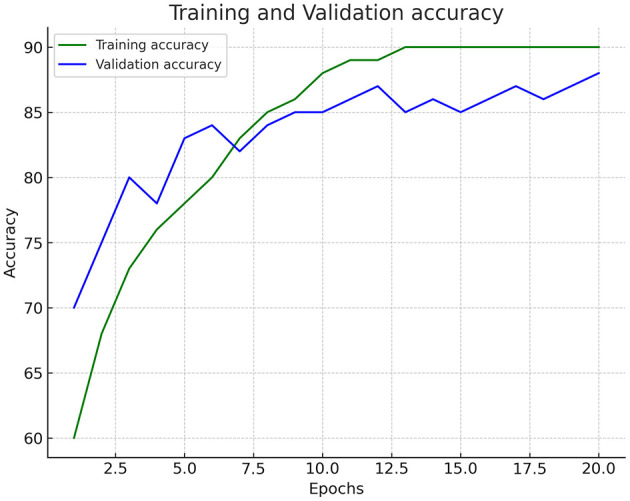
Training and validation accuracy.

**Figure 3:**
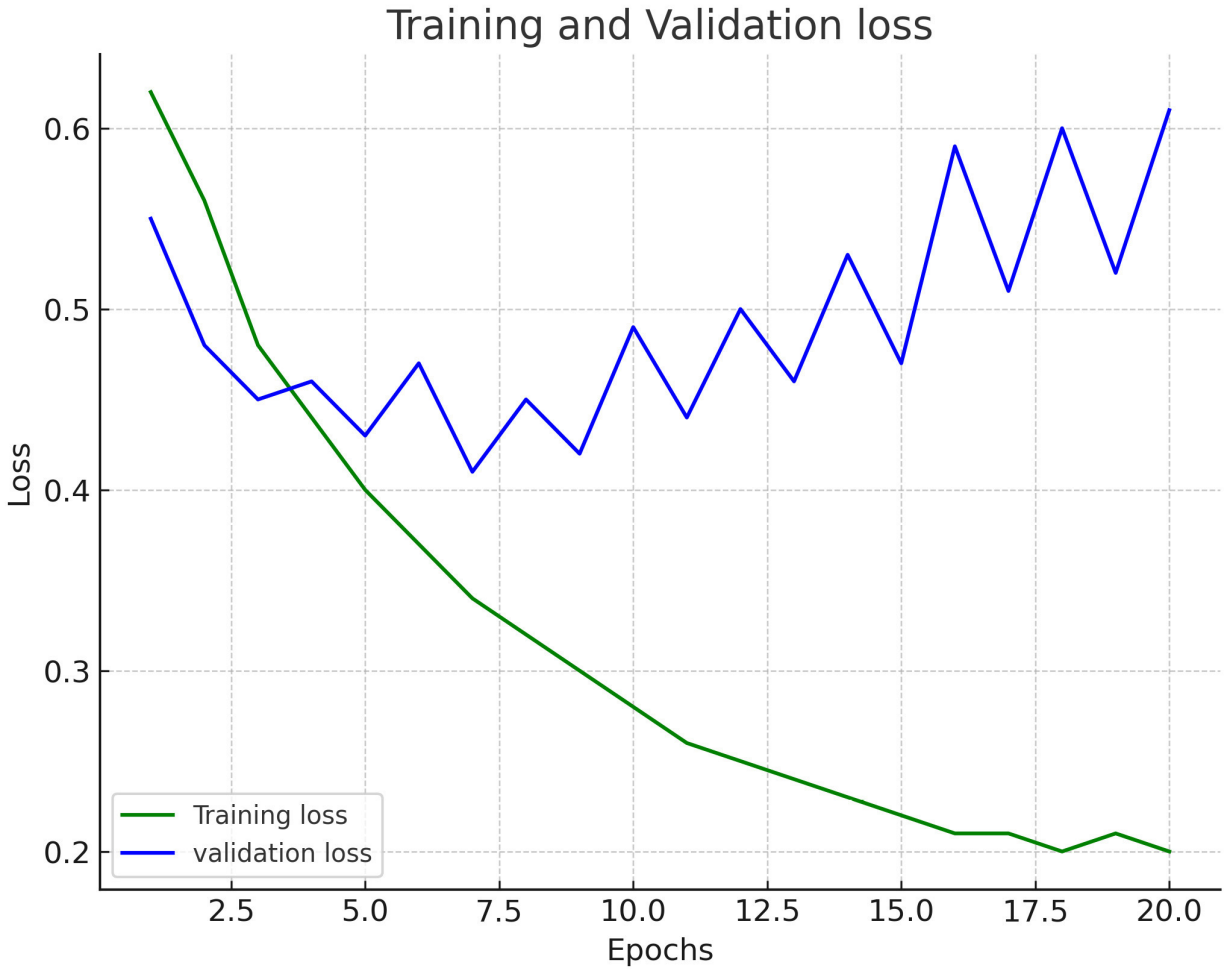
Training and validation loss.

Figure 2 shows the trained dataset's training and validation accuracy graph. While both the training accuracy and validation accuracy are increasing, the training accuracy is consistently higher than the validation accuracy. This could be a sign that the model is starting to overfit on the training data. The graph suggests that the model learns from the data and improves performance. However, it is essential to monitor the accuracy of the validation to avoid overfitting. The training and validation loss is increasing over time. Figure 3 shows the trained dataset's training and validation loss graph. This trend suggests that the model's performance worsens as the number of epochs increases, as shown in Table 2. It shows the hyperparameters utilized in the deepfake detection model, which includes a ResNet50 backbone and a two-layer LSTM for sequence modeling. The key parameters include a 224 × 224 input size, Adam optimizer, Binary Cross-Entropy loss, and regularization methods such as dropout (0.3) and L2 weight decay. In an ideal scenario, the training loss and validation loss would decrease as the number of epochs increases. This would signify that the model is learning from the training data and improving performance. An increase in loss indicates the opposite.

**Table 2:** Hyperparameters used in the Deepfake detection model.

**Hyperparameter**	**Value**
Backbone CNN	ResNet50 (Pre-trained on ImageNet)
Sequence model	LSTM (2 layers)
Input image size	224 × 224
Batch size	32
Learning rate	0.001 (with decay)
Optimizer	Adam
Loss function	Binary Cross-Entropy (BCE)
Dropout rate	0.3
LSTM hidden units	256
Number of epochs	10
Activation function	ReLU (CNN), Tanh (LSTM)
Pooling layer	Global Average Pooling (GAP)
Regularization	L2 weight decay (0.0001)
Early stopping	Patience = 3

### 4.1 Ablation studies

An ablation study is performed to evaluate the contribution of each component in our model by methodically eliminating or altering elements of the design. This facilitates comprehension of the influence of various ResNet backbones on performance, confirming that our suggested ResNet50 + LSTM model is the most efficacious for deepfake detection.

#### 4.1.1 Comparison between different ResNet pre-trained models

**Figure 4:**
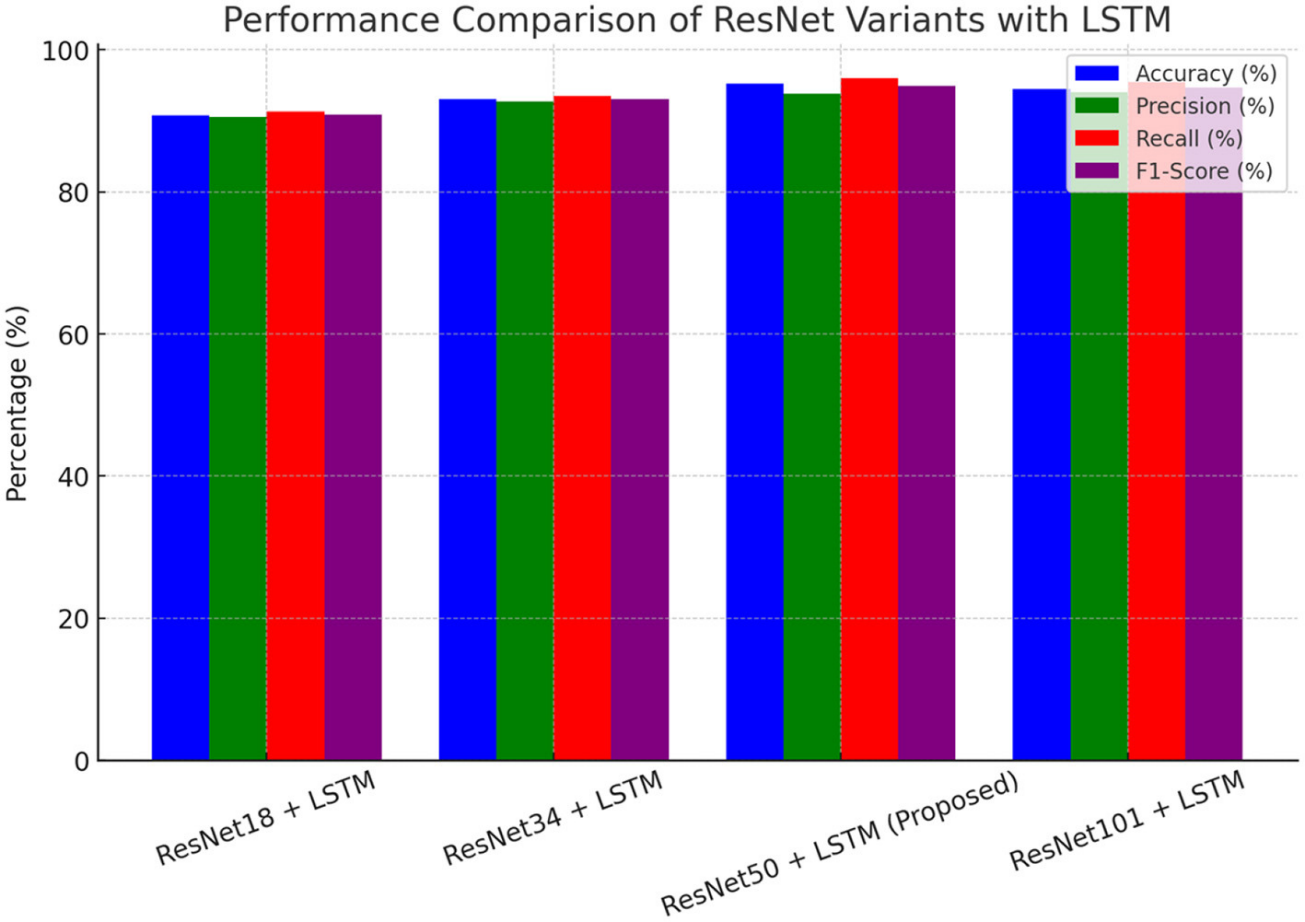
Comparative analysis of ResNet variants with LSTM.

Table 3 and Figure 4 illustrate the performance comparison of ResNet versions integrated with LSTM for deepfake detection. The assessment measures include accuracy, precision, recall, and F1-score. The findings demonstrate that an increase in the depth of the ResNet backbone correlates with enhanced model performance. The suggested ResNet50 + LSTM model attains an accuracy of 95.2%, surpassing models based on ResNet18 and ResNet34, while preserving a balance between computational efficiency and detection efficacy. The ResNet101 + LSTM model attains an accuracy of 94.5% but with heightened complexity. This indicates that ResNet50 effectively balances accuracy and computational expense, making it the ideal selection for deepfake detection in this research.

**Table 3:** Performance comparison of different ResNet variants with LSTM for deepfake detection.

**Model**	**Accuracy (%)**	**Precision (%)**	**Recall (%)**	**F1-Score (%)**
ResNet18 + LSTM	90.8	90.5	91.3	90.9
ResNet34 + LSTM	93.1	92.7	93.5	93.1
ResNet50 + LSTM (Proposed)	95.2	93.8	96.0	94.9
ResNet101 + LSTM	94.5	94.0	95.5	94.7

#### 4.1.2 Comparison of ResNet and EfficientNet CNN

This study on deepfake detection found that ResNet, particularly its deeper variants like ResNet-50, proved highly effective for high-resolution video analysis due to several key advantages in its design and performance. ResNet-50 was shown to excel in extracting intricate and nuanced features from high-resolution images due to its depth, which allows hierarchical representations to be learned effectively. This depth enabled the capture of complex patterns and textures in high-resolution frames, making subtle manipulations in video content more accessible to detect. ResNet's use of residual connections enhanced its ability to manage the complexities associated with significant and high-dimensional data inputs. These connections facilitated smoother gradient flow during training, mitigating issues such as vanishing gradients that can hinder the learning process in deep networks. This robustness is particularly beneficial when dealing with high-resolution videos' increased computational demands and memory requirements. ResNet's modular architecture was also noted to support efficient scaling, allowing significant inputs to be processed more effectively than architectures relying on complex scaling strategies like EfficientNet's compound scaling approach. In practical terms, ResNet's proven track record across various computer vision tasks underscores its reliability for deepfake detection in high-resolution videos.

**Figure 5:**
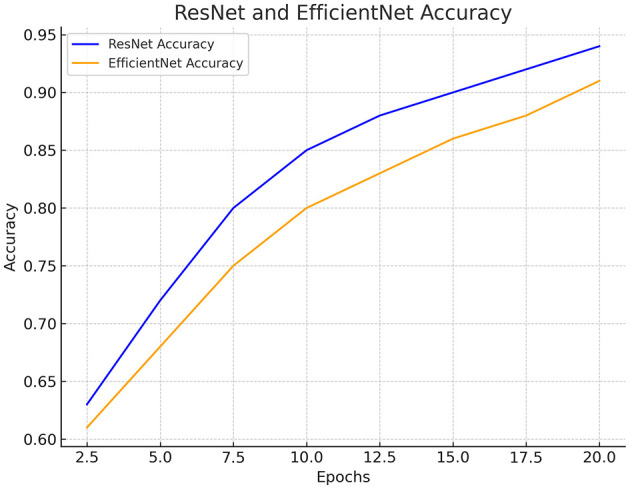
Comparison between ResNet and efficient CNN.

In contrast, ResNet's straightforward yet powerful architecture provides a stable foundation for handling high-resolution video data's intricate details and nuances, ensuring robust performance and accurate deepfake detection. In conclusion, the results demonstrate that ResNet stands out as a preferred choice for deepfake detection in scenarios involving high-resolution videos due to its depth, robust feature extraction capabilities, and efficient handling of significant inputs. As advancements continue in deep learning architectures and frameworks, ResNet's role remains pivotal in addressing the evolving challenges posed by sophisticated deepfake techniques in multimedia content analysis. Figure 5 compares ResNet and EfficientNet CNN for the trained dataset to detect actual and fake videos.

#### 4.1.3 Comparison between LSTM and recurrent neural network (RNN)

The work on deepfake detection found that Long Short-Term Memory (LSTM) networks offer several distinct advantages over traditional Recurrent Neural Networks (RNNs), making them particularly well-suited for this task. One of the primary benefits identified was the ability of LSTMs to handle long-term dependencies effectively. Standard RNNs struggle with learning long-term dependencies due to the vanishing gradient problem. Still, LSTMs overcome this issue through their unique cell state and gating mechanisms, including input, forget, and output gates. These mechanisms allow LSTMs to retain and utilize information over long sequences, which is crucial for deepfake detection, where understanding patterns across numerous frames is necessary.

Another advantage observed was the capability of LSTMs for selective memory. Unlike RNNs, which lack a mechanism to remember or forget information selectively, LSTMs can learn which input parts are essential and need to be remembered or forgotten. The gating mechanisms facilitate this selective memory, enabling LSTMs to focus on the critical parts of video frames. This ability to selectively remember significant features enhances the precision of deepfake detection by allowing the model to concentrate on relevant details while ignoring extraneous information.

**Figure 6:**
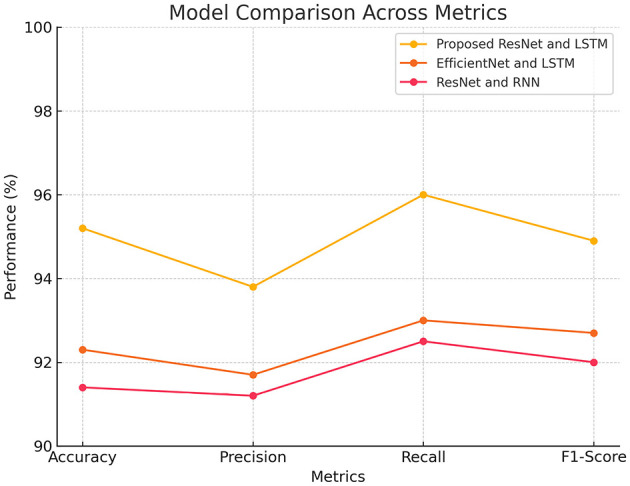
Comparison of the models.

It was also found that LSTMs exhibit better gradient flow compared to standard RNNs. In RNNs, issues with gradient flow during backpropagation through time can lead to vanishing or exploding gradients, hindering their ability to learn long-term dependencies effectively. LSTMs mitigate this problem with their internal structure, which supports better gradient flow and allows them to learn and adjust weights more effectively over long sequences. This improved learning capability is essential for tasks like deepfake detection, where capturing subtle temporal inconsistencies and anomalies in video sequences is necessary. Figure 6 compares the performance of three deep learning models: Proposed ResNet and LSTM, EfficientNet and LSTM, and ResNet and RNN, evaluated across four metrics: Accuracy, Precision, Recall, and F1-Score. The proposed ResNet and LSTM model regularly surpasses the other models accuracy, precision, and F1-score models. EfficientNet and LSTM exhibit strong performance; however, ResNet and RNN underperform, particularly in Accuracy and Precision. All models have high Recall scores.

## 5 Discussion

Furthermore, the work results indicated that LSTMs are more accurate in detecting deepfakes due to their ability to remember important information over extended periods and robust training mechanisms. Accurately detecting deepfakes often requires recognizing intricate patterns and inconsistencies spanning multiple frames. LSTMs' superior memory retention and selective focus on key features enable them to perform this task more precisely than standard RNNs. Lastly, it was found that LSTMs are more robust to noise compared to RNNs. Standard RNNs can be more susceptible to noise and irrelevant variations in the input data, which can degrade their performance. In contrast, the gating mechanisms in LSTMs help them filter out irrelevant information and focus on the key features needed to detect deepfakes accurately.

This robustness to noise ensures that LSTMs can maintain high performance even when the input data contains variability and extraneous details. We provide a visualization technique to improve model transparency by utilizing self-attention processes derived from transformer blocks. This technique elucidates how the model identifies deepfake material by producing temporal and geographical heatmaps that emphasize the focal regions during detection. The visualization-based interpretability provides valuable insights, showing where the model focuses during detection and offering explainable evidence of deepfake manipulations. The study results demonstrate that LSTMs significantly improve over standard RNNs in handling long-term dependencies, selective memory, gradient flow, accuracy, and robustness. These enhancements make LSTMs more effective for deepfake detection tasks, enabling them to capture subtle temporal inconsistencies and anomalies in video sequences with greater precision and reliability.

The proposed model efficiently integrates LSTM and ResNet architectures, showcasing its proficiency in reliably detecting deepfakes and aiding the formulation of powerful remedies against this widespread problem. The modular architecture guarantees adaptability and expandability, facilitating seamless integration into diverse platforms and applications. This approach underscores the significance of ethical dataset utilization, promoting responsible advancement and implementation of deepfake detection tools. The proposed approach establishes a robust basis for future research in this domain, facilitating the development of more sophisticated and efficient deepfake detection techniques. The deepfake detection method serves as an essential instrument for countering the proliferation of altered media and preserving the integrity of digital information. Current research in deepfake detection is moving in many directions to improve accuracy, robustness, and efficiency, especially concerning ResNet-LSTM-based models. Hybrid deep learning architectures are being investigated by merging sequence models like LSTMs and Transformers for temporal analysis with CNNs like ResNet and EfficientNet for feature extraction. Furthermore, a substitute for CNN-based feature extraction is Vision Transformers (ViTs).

Increasingly popular is multimodal deepfake detection, which enhances detection effectiveness by combining language patterns, physiological signs like heart rate and eye blinking, and audio-visual cues. The generalization of models across different manipulation approaches is still a significant difficulty, which has led to research into self-supervised learning, meta-learning techniques, and domain adaptability. One of its drawbacks is dependence on the caliber and variety of the training dataset. The model could have trouble generalizing to unseen deepfakes if the dataset lacks enough variability in deepfake methods or real-world distortions like occlusions, lighting conditions, and compression artifacts. Furthermore, real-time detection on devices with limited resources is difficult because of the increased computational complexity caused by the combination of ResNet and LSTM, even while it improves detection by collecting both spatial and temporal data. The possibility of adversarial assaults, in which deepfake generators constantly adapt to evade detection systems, is another drawback. For this to remain successful, regular model upgrades and retraining with fresh deepfake variants are required. Furthermore, since it takes a lot of time and effort to classify deepfake movies manually, the dependence on labeled datasets poses questions about scalability. The interpretability of the system is still a problem since deep learning models sometimes operate as “black boxes,” making it hard for users to comprehend the reasoning behind categorization choices.

## 6 Conclusion

Deepfake video detection using deep learning marks a significant advancement in protecting the integrity of digital media. Leveraging advanced neural network architectures like LSTM and ResNet makes it possible to distinguish between real and manipulated videos effectively. The proposed system adopts a comprehensive approach with two main flows: training and prediction. This involves preprocessing uploaded videos, splitting them into frames, and using a hybrid deep learning model to classify them as real or fake. Evaluating the model's performance through a confusion matrix ensures its reliability before deployment in practical applications. Despite the progress in this field, several challenges remain, highlighting areas for future research. Enhancing the model's ability to generalize across diverse datasets is crucial to ensure effectiveness against various deepfake techniques. Improving preprocessing methods, such as adaptive face detection and dynamic frame analysis, could increase accuracy. Additionally, developing real-time detection capabilities is essential to meet the rising demand for immediate verification in live streaming and video conferencing.

Future research should also explore the integration of multimodal data, combining audio and visual cues to improve detection accuracy and interpretability using advanced techniques. Developing adversarial training methods can improve the robustness of the model against increasingly sophisticated deep-fake generation techniques. Establishing standardized benchmarks and datasets for deepfake detection will facilitate consistent evaluation and comparison of different models, fostering collaboration and innovation within the research community. These efforts will help the field continue to progress, ensuring the integrity and reliability of digital media in the face of evolving deepfake technologies.

## Data Availability

Publicly available datasets were analyzed in this study. This data can be found at: https://www.kaggle.com/c/deepfake-detection-challenge/data.
